# An Assessment of Multipollutant Exposures Using Silicone Wristbands Among Bangladeshi Youth

**DOI:** 10.3390/ijerph21121691

**Published:** 2024-12-18

**Authors:** Margaret Quaid, Syed Emdadul Haque, Tariqul Islam, Mohammad Hasan Shahriar, Golam Sarwar, Alauddin Ahmed, Steven O’Connell, Farzana Jasmine, Muhammad G. Kibriya, Habibul Ahsan, Maria Argos

**Affiliations:** 1 Department of Environmental Health, School of Public Health, Boston University, Boston, MA 02118, USA; 2 UChicago Research Bangladesh, Dhaka 1230, Bangladesh; 3 Institute for Population and Precision Health, The University of Chicago, Chicago, IL 60637, USAfarzana@uchicago.edu (F.J.);; 4 MyExposome, Inc., Corvallis, OR 97333, USA; steven.oconnell@myexposome.com

**Keywords:** exposome, volatile organic compounds, silicone wristband personal monitoring, wearable sensor, Bangladesh, children, multipollutant

## Abstract

Residents of Bangladesh are exposed to numerous chemicals due to local industries, including dyeing mills, cotton mills, and the use of biomass in daily cooking. It is, therefore, important to characterize the exposome and work to identify risk factors of exposure. We used silicone wristband passive samplers to evaluate exposure to volatile and semi-volatile organic compounds in a sample of 40 children in the Araihazar upazila of Bangladesh. We used stepwise linear regression models to determine which demographic, exposure, diet, and socioeconomic factors best predict exposure to single chemicals and classes of chemicals. Male sex at birth was associated with a decrease in the number of chemicals detected above their median concentration (β = −2.42; 95%CI: −5.24, 0.399), as was ownership of a flush toilet (β = −3.26; 95%CI: −6.61, 0.097). Increased body mass index (β = 1.81; 95%CI: 0.587, 3.03), father’s smoking (β = 2.74; 95%CI: −0.0113, 5.49), and father’s employment in the garment industry (β = 3.14; 95%CI: 0.209, 6.07) were each associated with an increase in the average number of chemicals detected above their median concentration. The observed results motivate future evaluation with health outcomes of these exposures.

## 1. Introduction

Rural communities in Bangladesh encounter a unique mixture of chemicals from various sources in their daily lives. Some of these exposures have been well characterized in drinking water (e.g., inorganic arsenic) [1,2,3,4], but many other exposures and exposure sources are largely unexplored. Some aspects of these communities increase their likelihood of encountering certain chemicals. For example, nearly all the residents of rural Bangladesh use biomass—such as dried plants or animal dung—as cooking fuel [5]. These solid fuels can lead to exposure to fine particulate matter (PM2.5) containing polycyclic aromatic hydrocarbons (PAHs) and carbon monoxide [6,7,8]. Garment industry work is also prevalent in Bangladesh, which can lead to exposure to dyes [9]. Many studies have explored exposure to heavy metals/metalloids in these communities [1,4], but few characterized volatile and semi-volatile organic compound exposures, which may significantly contribute to health in the region [10,11].

A novel method for personal passive sampling makes the characterization of organic compound exposures possible. Silicone wristbands have been used in previous studies to examine relative exposures to a variety of chemicals; for example, Wang et al. used them to investigate semi-volatile organic chemical exposure in France and Italy, Doherty et al. used them to investigate chemical exposures in a birth cohort in New Hampshire, and McLarnan et al. investigated PAH exposure in a birth cohort based in New York [11,12,13]. They are worn by each participant for a predetermined length of time, then screened for up to 1528 chemicals, depending on the laboratory platform and chemicals of interest, that have diffused into the wristband both from the air and from the sweat of the participant [14,15,16]. This technology has been validated across many studies and allows for the external exposome of an individual to be quantified across the study period and compared to other individuals [17,18].

This method is particularly novel when applied to the Bangladeshi population. Rural regions of Bangladesh have limited access to electricity, making many other methods of characterizing the exposome in these regions unreliable. For this reason, there has been limited ability to study the exposome in resource-limited communities such as the one described in this study. The silicone wristband devices have made it possible to effectively characterize exposures that may be unique to the children who reside in this region of Bangladesh.

The resulting data can then be used to investigate the risk factors associated with exposure to certain chemicals and evaluate potential adverse health outcomes that may occur because of these exposures. For example, exposure to indoor particulate matter that arises from cooking with biomass fuel has been linked with asthma development, adverse cardiopulmonary outcomes, and adverse neurological symptoms [19,20]. Garment work is associated with a decreased immune response, which may result from occupational chemical exposures [21]. Some combination of these exposure sources may be responsible for the increased prevalence of chronic obstructive pulmonary disease, cardiovascular disease, diabetes, and chronic kidney disease in Bangladesh [22,23,24]. Therefore, characterization of the exposome of residents of rural Bangladesh can help to determine sources of toxic exposures and aid in identifying the most effective interventions to improve public health.

The goal of this pilot study was two-fold. First, we determined the prevalence of exposure to certain volatile and semi-volatile organic chemicals using silicone wristband passive sampling technology in a pediatric population of the Araihazar upazila (subdistrict) of Bangladesh. Second, we investigated the relationship between key demographic and lifestyle factors and exposure to the chemicals detected in the silicone wristbands. With this study, we enhance knowledge of the exposome of children residing in rural Bangladesh.

## 2. Materials and Methods

### 2.1. Study Population

The participants in this study are enrolled in the Bangladesh Environmental Research in Children’s Health (BiRCH) cohort, which is composed of the children of those enrolled in the Health Effects of Arsenic Longitudinal Study (HEALS). Both of these cohorts have been previously described [25,26]. Briefly, 11,746 married residents of the Araihazar upazila in Bangladesh were enrolled in the HEALS cohort between 22 October 2000, and 19 May 2002, and later expanded to include more than 35,000 participants. Between 2014 and 2016, 500 children of female participants in the HEALS cohort were enrolled in the BiRCH cohort as mother–child pairs. The children were between 5 and 7 years old at enrollment. In November 2021, forty children (20 male and 20 female) were randomly chosen to participate in this passive sampling study of semi-volatile organic compounds. This study was approved by the University of Illinois Chicago Office for the Protection of Research Subjects (protocol #2014-0408).

### 2.2. Covariate Data Collection

Covariate data were collected via an interview with the participant’s mother to quantify potential exposures that may have occurred during pregnancy or early childhood. Data were obtained to describe the demographic information, potential sources of exposure, diet, and socioeconomic status. Assigned sex at birth, age, height, weight, birth order, and health of the child were collected as descriptive factors. Measured height and weight data were used to calculate body mass index (BMI). The sources of exposure that were investigated included the smoking status of either parent, the smoking status of other individuals in the house, ownership of agricultural land, and paternal employment in the garment industry. Because garment/dye work is one of the main industries in Araihazar, a binary variable representing whether the parent worked in the garment industry was created. The following reported occupations were considered garment work: power loom, dyeing, garments, and cloth. To describe the diet of the child, the mother reported the child’s weekly consumption of the following items: rice, beans, fish, meat, vegetables, fruit, sweets, milk, juice, and water. Socioeconomic status was estimated from the following covariates: ownership of a television, type of toilet, and maternal educational attainment.

### 2.3. Exposure Data Collection

Silicone wristbands were obtained from 24 Hour Wristbands (Houston, TX, USA), rinsed with filtered water, and heated to at least 280 °C in a vacuum oven (Blue-M, New Columbia, PA, USA) at 0.1 torr for 3 h to remove any volatile impurities from the bands as previously described [12,14,27,28]. The participants were instructed to wear the wristband 24 h a day for a total of seven days, at which point the wristband was sealed into a polytetrafluoroethylene (PTFE) bag and stored at 4 °C at the field laboratory until all wristbands were returned. Wristbands were shipped overnight to MyExposome Inc. and stored at −20 °C until extraction.

MyExposome analyzed the wristbands used in this study for a total of 1528 chemicals [15,16]. To remove any particles and contaminants, the wristbands were cleaned before analysis with two rinses with 18 MΩ·cm of ultrapure water, then one rinse with isopropanol. They were then stored in amber glass jars at −20 °C until extraction could be performed. Chemicals were extracted sequentially into two 50 mL volumes of ethyl acetate. These two volumes were combined, and the sample was evaporated until 1 mL of solvent remained (Turbo-Vap L, Biotage, Charlotte, NC, USA) [12,14,27,28]. Samples were stored at 4 °C, then cleaned prior to analysis via solid phase extraction using acetonitrile and C18 columns to reduce analytical background and improve quantitative results and detection. The cleaned extracts were solvent exchanged into iso-octane and again stored at 4 °C until analysis could be completed.

To quantify the chemicals in the provided samples, MyExposome implemented gas chromatography (6890 N Gas Chromatograph, Agilent, Santa Clara, CA, USA) and mass spectrometry (5975B Mass Selective Detector), along with Automated Mass Spectral Deconvolution and Identification (AMDIS) software as described elsewhere, including reporting limits and analytical performance [16]. A matrix was produced containing the detected concentrations of each chemical found on each participant’s wristband. These concentrations were presented in units of nanogram chemical per gram wristband. If a chemical was not detected, a value of zero was assigned for that chemical.

Quality control samples were included to ensure results did not contain chemical detections or concentrations from the laboratory or the silicone itself. These quality control samples included blank unworn wristbands, solvent extraction blanks, instrument blanks, and continuing calibration verification samples to ensure instrument performance during analysis. Among all of the tested blanks, only 3 compounds were detected, bis(2-ethylhexyl) phthalate, diethyl phthalate and di-n-butyl phthalate. The highest content present among all blanks was background subtracted from all samples.

### 2.4. Statistical Analysis

Descriptive statistics were conducted and presented for the participants in this study. In the chemical exposure data, we imputed 0.0001 for nondetectable values for the purposes of log transformation and statistical analysis. Detection rates and summary statistics were calculated for every chemical detected in more than 50% of the study population. These chemicals were used in further analysis. Simple linear regression was performed to evaluate associations between sociodemographic and dietary variables with each chemical.

Stepwise linear regression models were constructed to determine which covariates best predicted the presence of the most widely detected chemicals in the wristbands. Each chemical that was detected in more than 50% of the participant samples was individually modeled as an outcome, with the following covariates investigated for inclusion in the model: assigned sex at birth, age, BMI, birth order, child’s overall health, parent smoking status, smoking status of other people in the house, whether the family owns agricultural land, paternal employment in the garment industry, diet variables, whether the family owns a television, toilet type, and maternal educational attainment. In models where sex at birth and/or age were not selected for inclusion, these variables were manually added back into the model. Models were additionally run replacing the BMI variable with the child’s height, the child’s weight, and the child’s height and weight as a sensitivity analysis.

In further analyses, we constructed a cumulative exposure score by summing the number of chemicals detected above their median level. This score was based only on chemicals that were detected in at least 50% of the samples. Stepwise models were performed, the outcome of which was the cumulative exposure score.

The chemicals were then divided into categories based on the MyExposome chemical class (e.g., PAH, chemicals in commerce, pesticide). For this purpose, one chemical could be represented in multiple classes. Within each chemical class, we made a count of the number of chemicals detected above their median level. For each class represented among the most commonly detected chemicals, a stepwise linear regression model was conducted with the covariates listed above to determine which factors best predict the exposure score for each chemical class. Assigned sex at birth and age were again manually added into each model.

Lastly, we performed principal component analysis (PCA) to reduce dimensionality. The chemical concentrations were log-transformed and scaled before PCA implementation. PCs with an eigenvalue greater than one were retained for analysis, and loadings were evaluated using a varimax rotation. Chemicals with a loading of more than 0.5 or less than −0.5 were considered to be represented by the principal component for the purposes of interpretation. The PCs were then individually modeled as the outcomes of interest using linear regression. All covariates described above were evaluated, and stepwise selection was implemented to determine which set of covariates best predicted the exposure to the given groupings of chemicals. These results were then compared to the findings in the single-chemical linear regression models to produce a more complete analysis of the demographic predictors correlated with these exposures. All analyses were performed in R (v. 4.2.1).

## 3. Results

### 3.1. Descriptive Statistics

Forty children participated in this study, 20 male and 20 female. They were between five and six years old at enrollment. Among this sample, 58% reported family ownership of agricultural land, which may indicate pesticide or herbicide exposure. While none of the mothers reported smoking, 48% reported paternal smoking, and 28% reported smoke exposure in the home by another smoker (Table 1). Approximately 33% reported paternal employment in the garment industry (Table 1). A full description of the cohort is reported in Table 1. For the purposes of comparison, we constructed a count of the number of chemicals detected above their median concentration in each wristband. We separately described those below the 25th percentile of exposure (based on chemical count) and those above the 75th percentile of exposure (Table 1). Participants exposed to a greater number of chemicals were more likely to be female, healthy, second-born, have a higher BMI, have a father who works in the garment industry, and have parents who own farmland than participants exposed to a fewer number of chemicals (Table 1). Participants exposed to a greater number of chemicals were less likely to have a non-parent smoker in the house or own a television as compared to participants with a fewer number of chemical exposures (Table 1).

### 3.2. Exposure Assessment

A total of 83 chemicals were detected in at least one study participant. Descriptive statistics for all 83 chemicals and the chemical class that they fall into are shown in Supplementary Table S1. Eighteen chemicals were present in only one wristband and eight chemicals were detected in only two wristbands. The median number of chemicals detected on a single wristband was 27.5 (interquartile range = 6.25; Table 2). PAHs were the chemical class most highly represented in the wristbands (median = 13 chemicals/wristband, interquartile range = 2; Table 2), followed by chemicals in commerce (median = 9 chemicals/wristband, interquartile range = 3; Table 2). One chemical, bis(2-ethylhexyl) phthalate (DEHP), was detected in every wristband (Table 3).

**Figure 1:**
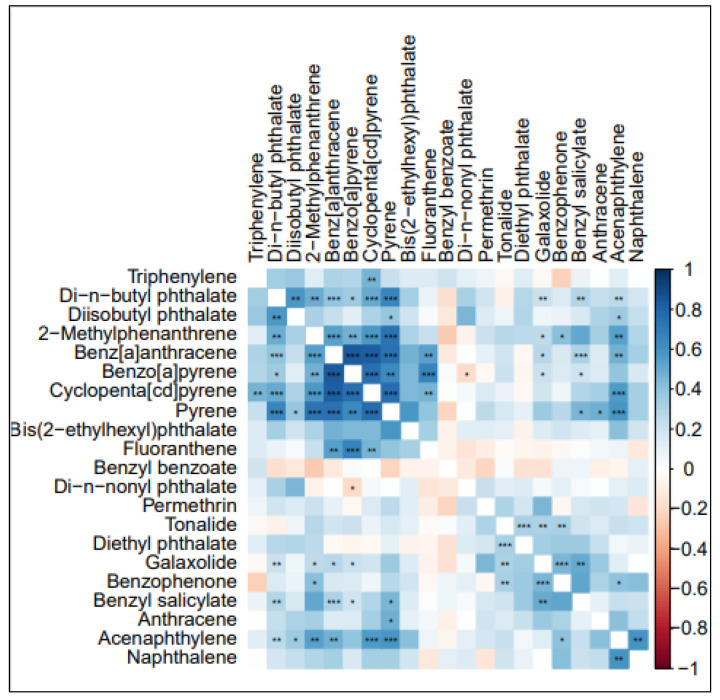
Spearman correlation plot of chemicals detected in ≥50% of wristbands. Blue indicates more positive correlation while red indicates negative. * *p*-value < 0.05; ** *p*-value < 0.01; *** *p*-value < 0.001.

For the purposes of these analyses, only the 21 chemicals that were detected in 50% or more of the wristbands were considered for further analyses. The median concentrations in wristbands of these chemicals are shown in Table 3, but it should be noted that these concentrations cannot be directly compared from chemical to chemical because they may diffuse into the wristband at different rates. Among this subset of 21 chemicals, PAHs (48%), chemicals in commerce (38%), and pesticides (24%) are the most common. Spearman correlations for the chemicals are shown in Figure 1, with the strongest correlations observed among PAHs.

### 3.3. Single Chemical Linear Regression

To characterize the associations between the demographic variables and the detected chemicals in each wristband, stepwise linear regression analyses were used. The number of predictors for each chemical ranged from five to nineteen, with tonalide being predicted by the fewest covariates (5 covariates, r^2^ = 0.203) and benzophenone being predicted by the highest number of covariates (19 covariates, r^2^ = 0.617, Supplementary Table S2). Some covariates appeared in prediction models more than others, with some occurring in as few as four models and some being featured in fifteen models. Vegetable consumption was included in the fewest models, while BMI was included in the most prediction models, apart from sex at birth and age which were manually included in each of the models (Supplementary Table S2). The R-squared of the prediction models ranged from 0.146 (fluoranthene) to 0.626 (diisobutyl phthalate; Supplementary Table S2).

We additionally investigated the total number of chemicals detected above their median concentration in each wristband, representing the overall cumulative exposure score (Table 4). Overall cumulative exposure score was positively associated with father’s smoking status and father’s garment work and inversely associated with socioeconomic status—as measured by television ownership and toilet type. We additionally found that male children were exposed to fewer chemicals than female children and that higher BMI was positively associated with the overall cumulative exposure score. Models in which height, weight, or height and weight were used in place of the BMI variable were not appreciably different from those constructed with the BMI variable and are reported in Supplementary Tables S3–S5.

### 3.4. Analysis by Chemical Classification

Class-specific cumulative exposure scores were also evaluated (Table 4). The number of chemicals detected above their median concentration in a given wristband ranged from 0 to 8 for commerce chemicals, 0 to 5 for personal care products, 0 to 4 for pesticides, 0 to 10 for PAHs, and 0 to 1 for volatile organic compounds (VOC). From stepwise linear regression models, we observed a similar pattern of results to the overall cumulative exposure score. In general, child BMI, father’s employment in the garment industry, and father’s smoking were positively associated with nearly every class-specific cumulative exposure score, whereas various measures of socioeconomic status—meat consumption, sweets consumption, juice consumption, television ownership, toilet type, or maternal educational attainment—were inversely associated with class-specific cumulative exposure score (Table 4).

### 3.5. PCA Analysis

**Figure 2:**
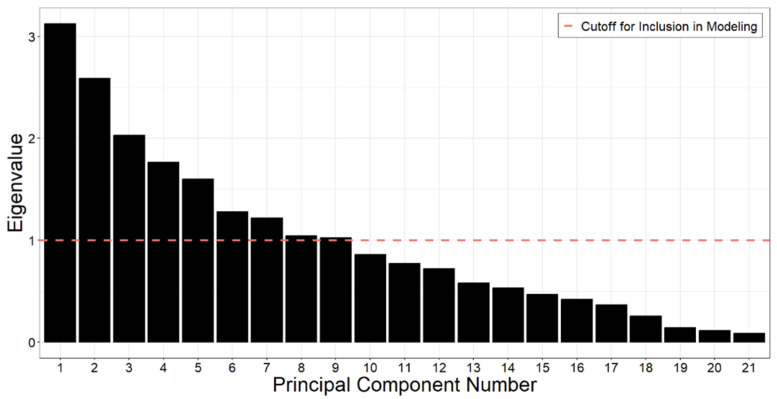
Eigenvalue associated with each principal component. Principal components with eigenvalues greater than 1 (red line) were used in the analysis. This included principal components 1 through 9.

Principal components (PC) analysis produced nine PCs that had eigenvalues greater than 1, which explain a cumulative 74.6% of the variance in the chemical detection dataset (Figure 2). Loadings are shown in Table 5.

**Figure 3:**
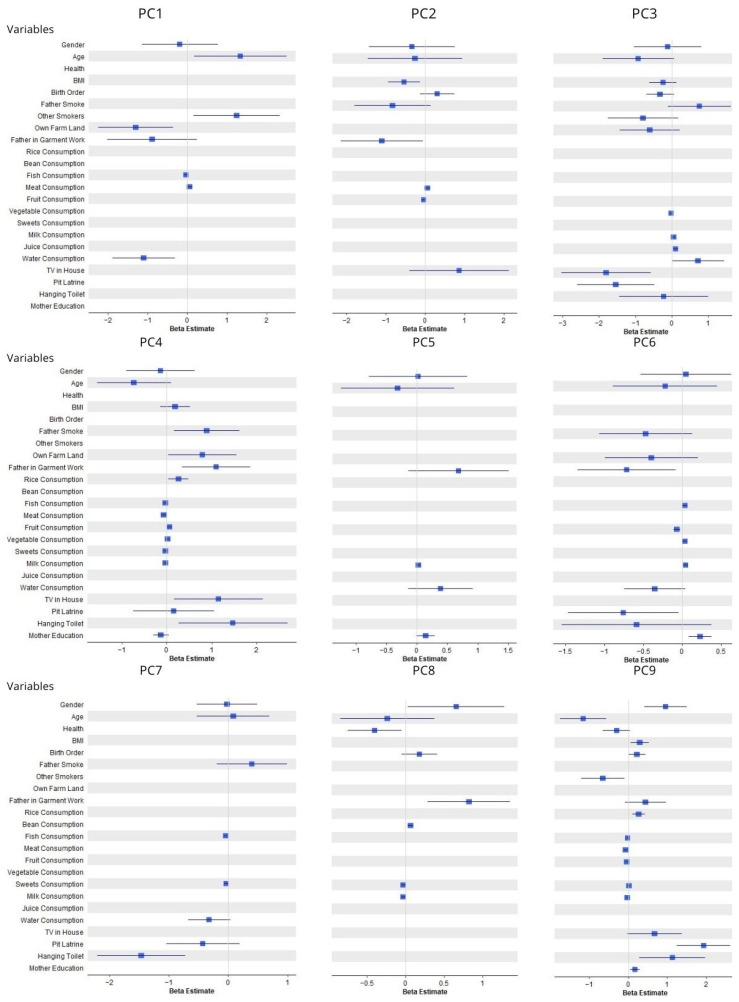
Principal components analysis results. Rice, beans, fish, meat, fruit, vegetables, sweets, milk, and juice consumption are all reported in units of days the food was consumed per month. Water consumption is reported in units of L/day.

Results from stepwise linear regression modeling for each PC are summarized in Figure 3. Again, similar predictors consistently emerged in these analyses. Father’s smoking was associated with PC2, PC3, PC4, PC6 and PC7. Father’s employment in the garment industry was associated with PC1, PC2, PC4, PC5, PC6, PC8 and PC9. Various proxies of socioeconomic status were associated with PC3, PC4, PC6, PC7, and PC9.

## 4. Discussion

This study presents novel data on the prevalence and patterns of organic chemical exposures among children residing in a rural community in Bangladesh. We observed that exposures to PAHs, chemicals in commerce, and pesticides were more prevalent than other chemical classes. We assessed various constructs of exposure patterns and observed consistent positive associations between father’s smoking status and father’s employment in the garment industry with higher exposure prevalence, whereas socioeconomic status was generally associated with lower exposure prevalence. Child characteristics, including female sex at birth and higher BMI, were also generally associated with higher exposure prevalence.

Many dietary variables were included in various models, though they were associated with very small effect sizes, suggesting that they were included to improve the variance of the models rather than because they were strong predictors. This is not surprising because, in many cases, the consumed food item is unlikely to directly affect VOC and SVOC chemicals detected on the wristbands in this cohort. One exception is that meat and vegetables may reflect exposures resulting from cooking these foods. Because much of the cooking is done using biomass, consumption of food that requires cooking on the stove can lead to increased exposure to PAHs [5,6,7,8]. Some of the other diet variables may be acting as a proxy for socioeconomic status, such as consumption of sweets, meat, and juice.

Benz(a)anthracene was detectable in all but one participant sample. Compared to other silicone wristband sampling studies, the prevalence observed in our study was notably higher [30,31]. This can be attributed to the widespread use of biomass fuel for cooking, as benz(a)anthracene is a known PAH resulting from biomass burning [32].

We identified several previous studies investigating children’s chemical exposure using passive wristband samplers. One study focused on PAH exposure in 24 children in Uruguay [33]. This study found a much broader variety of PAHs than our study, with 18 PAHs being detected in more than 50% of tested wristbands compared to the 10 found in this study. However, the concentrations they detected were much lower, with the highest median concentration observed being 16.3 ng/g wristband, while the highest median concentration for a PAH in our study was 175.5 ng/g wristband. Only two of the ten PAHs highly detected in our study had a lower median concentration than the participants in the Uruguay study, suggesting that the children in our study were more highly exposed to PAHs than the children studied in Uruguay. Some of the PAHs detected in the Uruguay study were not detected in our Bangladesh population. This finding may be due to a difference in the limit of detection between this study and our study. Targeted exposure studies have lower limits of detection than untargeted studies, such as our study [16]. It may be that the undetected PAHs were in fact present in our study, but at levels below the limit of detection.

Additionally, a few passive wristband studies have been conducted that focus on other pediatric exposures in communities. Many of these studies focus on organophosphates (OP), commonly used as flame retardants and pesticides [27,34,35,36]. Targeted studies conducted in Uruguay (sample size N = 24), Oregon (sample size N = 92), New York (sample size N = 64), and South China (sample size N = 94) each investigate these exposures and, in all cases, detect OPs in more than 50% of their participants, while we did not widely observe any of these chemicals. This suggests that the children in Araihazar may be less exposed to this type of chemical than children in other places around the world [27,34,35,36]. Alternatively, this finding may be explained by the targeted nature of these studies: the children in our study may have been exposed to OPs, but at levels below the limit of detection of the broader chemical analysis.

Some studies focused on other pesticides and generally found different exposure profiles than those identified in our current study. In one targeted study conducted in 10 children in North Carolina, permethrin was detected in only 40% of the participants compared to 87.5% in our study, and a targeted study conducted with 38 children in South Africa detected both deltamethrin and chlorpyrifos at high rates, while we did not detect any deltamethrin, and we detected chlorpyrifos in only 7.5% of participants [37,38].

One study targeting only phenol exposure in 203 residents of North Carolina detected many more parabens than were present in our current study, including methyl- and ethylparaben, which we did not detect in any participant [39]. There was one study that targeted nicotine and tobacco by-products which found nicotine at high levels in children whose parent smokes, while it was not detected at all in our present study [40]. These different exposure profiles among children indicate that the chemical exposures faced by children in Bangladesh may be substantially different from their peers in other countries, suggesting that this region is in need of further study.

One previous wristband passive sampling study was conducted in an adult Bangladeshi population, specifically in Dhaka’s e-waste dismantlers (N = 15) [11]. This study investigated exposure to brominated diphenyl ethers (BDEs), novel brominated flame retardants, dechlorane plus, and organophosphate esters, and they found phthalates and BDEs at very high detection rates [11]. Our current study showed very low detection rates for these types of chemicals, and the differences are likely because the Dhaka study was targeted towards BDEs and was conducted in an occupational cohort, while the present study was conducted in a community-based cohort of children. The findings of the current study represent an approximation of the community exposures that are encountered in the region, though they may be affected by the prevalence of the garment industry.

This study has some limitations. First, the small sample size limited the power of our analysis and the ability to conduct more nuanced statistical analysis. Second, the passive wristband samplers cannot directly attribute an exposure to any given source or behavior, and therefore, if there is an important exposure source that was not explored in the questionnaire, we may have missed important contributors. Third, we cannot compare concentrations between different chemical species within the same wristband because the chemicals may differ in their ability to absorb into the wristbands. Fourth, the questionnaire data were collected upon enrollment, which was five to seven years before the wristbands were distributed and may have changed. Lastly, caution must be used when comparing the findings from this study to other studies because much of the existing literature consists of targeted exposure investigations, with lower limits of detection for the specific chemicals investigated.

The use of passive wristband samplers was a strength of this study because they represent a very noninvasive way of collecting personal data on volatile and semi-volatile exposures and we were able to quantify a variety of chemicals in these wristbands. This technology provided the opportunity to evaluate exposures of children in a rural region of Bangladesh, with limited and inconsistent access to electricity. Recent scientific evidence suggests that wristband sampling devices correlate well with some serum biomarkers, SVOC concentrations in dust, and other passive samplers [30,39,41]. Additionally, because of the timing of the questionnaire data collection, we were able to take a prospective viewpoint of the factors that may result in exposure.

The purpose of this study was to characterize the exposome of the children in the Araihazar region of Bangladesh. These children are highly exposed to arsenic through their water and have higher risk of diabetes, chronic obstructive pulmonary disease, chronic kidney disease and adverse cardiovascular outcomes as they age [22,23,24]. These outcomes cannot be fully explained by arsenic exposure, so evaluating other chemical exposures is crucial to investigating whether other environmental exposures may be responsible for these increased risks of adverse health outcomes. The findings of this study will be implemented to evaluate the impact of these exposures on the health of the children studied.

## 5. Conclusions

Overall, this study presents novel information on patterns of organic chemical exposures in children residing in rural Bangladesh. The most prevalent exposure classes included PAHs, chemicals in commerce, and pesticides. Father’s smoking status and employment in the garment industry were consistently observed to be associated with exposure prevalence, whereas television ownership, toilet type, and mother education (all proxies of socioeconomic status) were observed to be inversely associated with exposure prevalence. In the future, the results acquired here may be used to evaluate the relationship between these exposures and adverse health outcomes.

## Figures and Tables

**Table 1:** Descriptive statistics stratified by the number of chemicals detected above the mean.

Characteristic	Full Cohort	Lower Exposed Participants	Higher Exposed Participants
	N = 40 ^1^	N = 10 ^1^	N = 12 ^1^
Gender			
Female	20 (50%)	5 (50%)	9 (75%)
Male	20 (50%)	5 (50%)	3 (25%)
Child age (years)			
5	11 (28%)	3 (30%)	2 (17%)
6	29 (73%)	7 (70%)	10 (83%)
Body Mass Index	14.34 (13.54, 15.19)	13.38 (12.96, 13.88)	15.11 (13.90, 16.05)
Birth Order			
1	10 (25%)	3 (30%)	1 (8.3%)
2	14 (35%)	2 (20%)	7 (58%)
3	8 (20%)	2 (20%)	2 (17%)
4	3 (7.5%)	1 (10%)	1 (8.3%)
5	5 (13%)	2 (20%)	1 (8.3%)
Health			
Almost Always Unwell	6 (15%)	3 (30%)	1 (8.3%)
Sometimes Quite Ill	3 (7.5%)	1 (10%)	0 (0%)
Healthy	31 (78%)	6 (60%)	11 (92%)
Very Healthy	0 (0%)	0 (0%)	0 (0%)
Mother Smokers			
Yes	0 (0%)	0 (0%)	0 (0%)
No	40 (100%)	10 (100%)	12 (100%)
Father Smokes			
Yes	19 (48%)	4 (40%)	6 (50%)
No	21 (53%)	6 (60%)	6 (50%)
Other Smokers in the House			
Yes	11 (28%)	4 (40%)	3 (25%)
No	29 (73%)	6 (60%)	9 (75%)
Own Farm Land			
Yes	23 (58%)	4 (40%)	9 (75%)
No	17 (43%)	6 (60%)	3 (25%)
Father Employed in Garment Industry			
Yes	13 (33%)	1 (10%)	6 (50%)
No	27 (68%)	9 (90%)	6 (50%)
Television Ownership			
Yes	32 (80%)	8 (80%)	6 (50%)
No	8 (20%)	2 (20%)	6 (50%)
Toilet Type			
Pit Latrine	21 (53%)	7 (70%)	5 (42%)
Hanging Toilet	9 (23%)	1 (10%)	3 (25%)
Flush	10 (25%)	2 (20%)	4 (33%)
Maternal Educational Attainment			
No formal education	2 (5%)	1 (10%)	0 (0%)
Up to primary	11 (28%)	3 (30%)	2 (17%)
Secondary school certificate	25 (63%)	5 (50%)	9 (75%)
Higher secondary certificate or higher	0 (0%)	0 (0%)	0 (0%)
Unknown	2 (5%)	1 (10%)	1 (8%)

^1^ N (%); Median (Q1, Q3). For each participant, we constructed a count of the number of chemicals detected above their median concentration (from the study population). Participants whose count was below or equivalent to the 25th percentile were considered “lower exposed” in this table, and those who were in the 75th percentile of exposure were considered “higher exposed”.

**Table 2:** Statistics of chemicals per wristband.

Chemical	Median (25th Percentile, 75th Percentile)	Range
Total	27.5 (23, 29.25)	(14, 37)
Chemicals in Commerce	9 (8, 11)	(5, 14)
Consumer Products	1 (0, 1)	(0, 4)
Dioxins and Furans	0 (0, 0)	(0, 1)
Flame Retardant	0 (0, 1)	(0, 2)
Personal Care	6 (4, 7)	(1, 11)
Pesticides	6 (5, 7)	(3, 9)
Pharmacological	0 (0, 0)	(0, 1)
Polycyclic Aromatic Hydrocarbons (PAHs)	13 (12, 14)	(5, 20)
Volatile Organic Compounds (VOC)	1 (1, 2)	(0, 2)

Chemical classes were assigned by MyExposome Inc.

**Table 3:** Chemicals (ng/g silicone) detected in wristbands in at least 50% of study samples.

Chemical	CASN	CID	% Detected	Median (25th and 75th Percentile)	MyExposome Classification
Bis(2-ethylhexyl)phthalate	117-81-7	8343	100	1540 (874, 2360)	Chemicals in Commerce and Pesticides
Benz[a]anthracene	56-55-3	5954	97.5	28.2 (20.8, 34.9)	Polycyclic Aromatic Hydrocarbons
Pyrene	129-00-0	31,423	97.5	176 (133, 207)	Chemicals in Commerce and Polycyclic Aromatic Hydrocarbons
Acenaphthylene	208-96-8	9161	95	17.8 (12.5, 24.8)	Polycyclic Aromatic Hydrocarbons
Benzyl salicylate	118-58-1	8363	95	374 (170, 822)	Personal Care
Diisobutyl phthalate	84-69-5	6782	95	1260 (601, 2960)	Chemicals in Commerce
Diethyl phthalate	84-66-2	6781	92.5	436 (238, 1090)	Chemicals in Commerce and Pesticides
Naphthalene	91-20-3	931	92.5	11.6 (9.04, 16)	Polycyclic Aromatic Hydrocarbons and Volatile Organic Compounds
Di-n-nonyl phthalate	84-76-4	6787	90	328 (177, 572)	Chemicals in Commerce
Permethrin	52645-53-1	40,326	87.5	207 (92.4, 584)	Pesticides
Di-n-butyl phthalate	84-74-2	3026	85	3080 (618, 9200)	Chemicals in Commerce, Personal Care, and Pesticides
Benzo[a]pyrene	50-32-8	2336	82.5	18.6 (10.5, 22.2)	Polycyclic Aromatic Hydrocarbons
Anthracene	120-12-7	8418	77.5	124 (39.7, 183)	Polycyclic Aromatic Hydrocarbons
Cyclopenta[cd]pyrene	27208-37-3	33,743	77.5	59.1 (30.2, 82.1)	Polycyclic Aromatic Hydrocarbons
2-Methylphenanthrene	2531-84-2	17,321	72.5	17.4 (0, 23.2)	Polycyclic Aromatic Hydrocarbons
Fluoranthene	206-44-0	9154	70	152 (0, 202)	Polycyclic Aromatic Hydrocarbons
Galaxolide	1222-05-5	91,497	70	91.8 (0, 279)	Chemicals in Commerce and Personal Care
Benzophenone	119-61-9	3102	65	21.7 (0, 30.4)	Chemicals in Commerce and Personal Care
Tonalide	1506-02-1	89,440	60	29.4 (0, 61.6)	Personal Care
Triphenylene	217-59-4	9170	52.5	9.48 (0, 30.4)	Polycyclic Aromatic Hydrocarbons
Benzyl benzoate	120-51-4	2345	50	18.9 (0, 176)	Pesticides

CID: PubChem Compound Identifier [29].

**Table 4:** Stepwise modeling of predictors in relation to overall and class-specific cumulative exposure score.

	Class-Specific Cumulative Exposure Score (Beta Coefficient and 95% Confidence Interval)					Overall Cumulative Exposure Score
	Commerce	Personal Care Product	Pesticide	PAHs	VOCs	
Male Sex at Birth	−1.05 (−2.48, 0.37)	−1.07 (−1.79, −0.344)	−0.838 (−1.51, −0.166)	−1.23 (−3.18, 0.73)	0.0135 (−0.325, 0.351)	−2.42 (−5.24, 0.399)
Age	0.279 (−1.13, 1.69)	0.933 (0.0836, 1.78)	0.107 (−0.734, 0.948)	0.563 (−1.93, 3.05)	0.0983 (−0.265, 0.461)	1.39 (−2.08, 4.86)
Child Health		−0.528 (−1.07, 0.0113)				
BMI	0.831 (0.349, 1.31)	0.718 (0.381, 1.06)	0.358 (0.0788, 0.637)	1.01 (0.152, 1.87)	0.114 (−0.00212, 0.230)	1.81 (0.587, 3.03)
Birth Order	−0.332 (−0.830, 0.167)				−0.129 (−0.262, 0.00435)	
Father Smoke	0.710 (−0.356, 1.78)	0.540 (−0.160, 1.24)		1.87 (−0.170, 3.92)		2.74 (−0.0113, 5.49)
Other Smokers				−2.09 (−4.39, 0.215)		−2.36 (−5.43, 0.707)
Own Farm Land		0.610 (−0.206, 1.43)				
Garment	0.776 (−0.394, 1.95)	0.500 (−0.247, 1.25)	0.652 (−0.0657, 1.37)	2.23 (0.153, 4.31)		3.14 (0.209, 6.07)
Rice			−0.141 (−0.323, 0.0407)	0.700 (0.130, 1.27)		
Beans	0.0764 (0.013, 0.140)	0.0402 (−0.00376, 0.0842)	0.0397 (0.00102, 0.0783)			0.115 (−0.0365, 0.267)
Meat					0.0133 (−0.00182, 0.0284)	−0.166 (−0.367, 0.0355)
Vegetables	−0.0657 (−0.146, 0.0148)		−0.0591 (−0.109, −0.00940)	−0.0799 (−0.219, 0.0593)		0.0859(−0.0434, 0.215)
Sweets	−0.0502 (−0.109, 0.00886)	−0.0331 (−0.0735, 0.00724)	−0.0320 (−0.0694, 0.00550)			−0.113 (−0.27, 0.0443)
Milk		−0.0302 (−0.0652, 0.00474)	−0.0344 (−0.0685, −0.000298)			
Juice		−0.0885 (−0.151, −0.0257)		−0.119 (−0.284, 0.0463)		−0.179 (−0.418, 0.0591)
Water		−0.499 (−1.06, 0.0569)				
TV in House	−1.92 (−3.36, −0.474)					−2.95 (−6.52, 0.628)
Hanging Toilet		−0.442 (−1.42, 0.535)		−2.13 (−5.04, 0.793)		−3.1 (−7.05, 0.852)
Flush		−0.815 (−1.71, 0.0752)		−3.62 (−6.25, −0.979)		−3.26 (−6.61, 0.0967)
Mother Education	−0.147 (−0.358, 0.0645)				0.0465 (−0.0105, 0.104)	
R-Squared	0.471	0.529	0.366	0.217	0.246	0.411

Abbreviations: Polycyclic aromatic hydrocarbons (PAHs) and volatile organic compounds (VOCs). Reported values are beta coefficients (95% confidence interval). This portion of the analysis included only the 21 chemicals there were detected in at least 50% of the participants. The median was calculated from only the wristbands where the chemicals were detected. Rice, beans, fish, meat, fruit, vegetables, sweets, milk, and juice are recorded as the number of days per month that the child consumes the food. Water consumption is recorded in units of L/day.

**Table 5:** Varimax rotated loadings for principal component analysis.

	PC1	PC2	PC3	PC4	PC5	PC6	PC7	PC8	PC9
Bis(2-ethylhexyl)phthalate	−0.018	−0.240	−0.193	0.049	0.110	−0.231	−0.002	**0.668**	0.247
Benz[a]anthracene	−0.126	−0.048	0.071	0.055	−0.132	0.174	**0.764**	0.010	0.113
Pyrene	−0.051	−0.024	−0.038	−0.070	−0.018	**−0.854**	−0.011	0.050	0.089
Acenaphthylene	0.036	−0.139	0.006	−0.023	**0.739**	0.002	0.053	0.056	0.068
Benzyl salicylate	−0.111	0.052	0.021	**−0.894**	0.020	−0.036	−0.038	−0.181	0.038
Diisobutyl phthalate	0.078	−0.103	0.340	**−0.810**	−0.023	−0.110	0.005	0.107	−0.064
Diethyl phthalate	0.018	−0.268	0.073	−0.001	−0.201	0.321	**−0.676**	−0.034	0.093
Naphthalene	−0.183	−0.078	−0.112	0.039	**0.770**	−0.025	−0.018	0.109	−0.107
Di-n-nonyl phthalate	0.209	0.154	0.009	−0.023	−0.156	0.001	0.021	−0.289	**−0.780**
Permethrin	**0.514**	−0.015	0.324	0.187	−0.018	−0.074	0.032	−0.483	−0.285
Di-n-butyl phthalate	0.423	−0.226	**0.555**	−0.148	−0.062	−0.089	0.042	−0.032	−0.024
Benzo[a]pyrene	−0.056	−0.105	0.324	−0.031	0.390	0.022	0.081	**0.688**	−0.188
Anthracene	**0.800**	0.196	−0.152	0.105	−0.065	0.002	−0.098	0.074	−0.067
Cyclopenta[cd]pyrene	0.060	−0.489	0.162	−0.055	0.177	0.033	**0.652**	0.151	0.057
2-Methylphenanthrene	−0.172	**−0.865**	0.050	−0.094	0.199	−0.010	0.031	0.086	−0.096
Fluoranthene	−0.222	0.354	−0.002	0.161	−0.362	0.144	0.315	**0.584**	−0.035
Galaxolide	**0.765**	−0.041	0.000	−0.128	−0.096	0.079	−0.042	−0.173	−0.101
Benzophenone	0.270	−0.309	−0.448	**−0.584**	0.105	0.256	−0.005	0.258	−0.025
Tonalide	0.056	−0.300	−0.018	−0.007	0.185	0.119	−0.125	0.170	**−0.815**
Triphenylene	−0.185	0.039	**0.864**	−0.138	−0.009	0.152	0.071	0.011	0.025
Benzyl benzoate	−0.286	0.446	0.203	−0.147	**0.520**	0.357	0.146	−0.068	0.104
Eigenvalue	3.1	2.6	2	1.8	1.6	1.3	1.2	1	1
Variance Percent Explained	14.9%	12.3%	9.7%	8.4%	7.6%	6.1%	5.8%	5.0%	4.9%
Cumulative Variance Explained	14.9%	27.2%	36.9%	45.3%	52.9%	59.0%	64.8%	69.8%	74.6%

**Bolded values** indicate that they loaded more than 50% onto the principal component, either positively or negatively. They are considered to be included in that principal component for the purposes of analysis.

## Data Availability

The datasets generated and/or analyzed during the current study are not publicly available due to privacy protections but are available from the corresponding author on reasonable request.

## Supplementary Materials

The following supporting information can be downloaded at: https://www.mdpi.com/article/10.3390/ijerph21121691/s1, Table S1: Chemicals Detected in Wristbands; Table S2: Results from Stepwise Modeling with Single Chemical Outcomes; Table S3: Results from sensitivity analyses replacing BMI with height; Table S4: Results from sensitivity analyses replacing BMI with weight; Table S5: Results from sensitivity analyses replacing BMI with height and weight.
